# Prognostic effect of parotid area lymph node metastases after preliminary diagnosis of nasopharyngeal carcinoma: a propensity score matching study

**DOI:** 10.1002/cam4.1154

**Published:** 2017-09-06

**Authors:** Yuanji Xu, Xiaolin Chen, Mingwei Zhang, Youping Xiao, Jingfeng Zong, Qiaojuan Guo, Sufang Qiu, Wei Zheng, Shaojun Lin, Jianji Pan

**Affiliations:** ^1^ Department of Radiation Oncology Fujian Cancer Hospital Fujian Medical University Cancer Hospital Fuzhou Fujian China; ^2^ Department of Radiation Oncology NO.2 Hospital Xiamen Xiamen Fujian China; ^3^ Department of Radiotherapy First Affiliated Hospital of Fujian Medical University Fuzhou Fujian China; ^4^ Department of Radiology Fujian Cancer Hospital Fujian Medical University Cancer Hospital Fuzhou Fujian China; ^5^ Fujian Provincial Key Laboratory of Translational Cancer Medicine Fuzhou Fujian China

**Keywords:** Nasopharyngeal carcinoma, parotid lymph node metastasis, prognosis, propensity score matching

## Abstract

Parotid area lymph node (PLN) metastasis in nasopharyngeal carcinoma (NPC) is rare, and its prognosis remains largely unknown. Our study aimed to investigate the prognostic value and staging categories of PLN metastasis in patients with NPC and treated with intensity‐modulated radiation therapy (IMRT), to provide a reference for clinical treatment for NPC with PLN metastasis. Records for 1616 untreated NPC patients without distant metastasis was retrospectively reviewed. All patients underwent magnetic resonance imaging (MRI) examination prior to treatment and then received IMRT as their primary treatment. Forty‐five NPC patients (2.8%) showed initial PLN metastasis on follow‐up MRI. PLN metastasis was significantly associated with the N classification and clinical stage. Univariate analysis showed that PLN metastasis had an unfavorable influence on overall survival (OS), progression‐free survival (PFS), distant metastasis‐free survival (DMFS), and regional relapse‐free survival (RRFS) in NPC patients. Using propensity score matching (PSM) to calibrate selection bias and confounding bias, it was observed that PLN metastasis remained an adverse prognostic factor for OS, PFS, DMFS, and RRFS. Furthermore, the 5‐year DMFS and RRFS curves for PLN metastasis were significantly separated from that for N2 disease but crossed that for N3 disease. Therefore, PLN metastasis was found to be an adverse prognostic factor for NPC and to be associated with the same DMFS as N3 disease. Therefore, more aggressive therapeutic strategies consistent with those for N3 disease are recommended for NPC with PLN metastasis to reduce distant metastasis.

## Introduction

Nasopharyngeal carcinoma (NPC) is the most commonly diagnosed head and neck malignancy in Southeast Asia, with unique aggregation in Southern China. Although 85% of cases of preliminarily diagnosed NPC involve cervical lymph node metastasis 1, parotid area lymph node (PLN) metastasis is rare, with an incidence rate of only 1–3.4% 1, 2, 3, 4, 5, 6. In addition, the PLN is generally regarded as an unconventional surgical neck dissection area 7. Furthermore, no case of PLN recurrence or metastasis with NPC was reported in the previous conventional radiotherapy era, as the bilateral parotid glands and surrounding lymph nodes were always covered by the definitive doses for the primary tumor. Therefore, little attention has been given to PLN metastasis in patients preliminarily diagnosed with NPC to date.

With the advent of intensity‐modulated radiation therapy (IMRT), not only were radiation doses to the primary tumor improved, but also the adjacent normal tissues were better protected 8. As such, the parotid gland area receives only low radiation doses to reduce the xerostomia and further improve the quality of life of patients with NPC 9, 10, 11, 12. However, subsequent studies have demonstrated that overprotection of the parotid gland resulted in a few cases of PLN recurrence after treatment of NPC with IMRT 13, 14, 15, which suggested that potential or definite PLN metastasis in preliminarily diagnosed patients might be neglected in clinical practice. Hence, the potential risk of initial PLN metastasis in patients with NPC receiving IMRT has garnered attention 6, 15. According to the latest guidelines for neck node level delineation in head and neck cancers 16, the parotid node group has been classified as level VIII.

However, the prognosis of NPC with initial PLN metastasis in patients with NPC in the IMRT era has not been well documented. Zhang et al. 4 reported the first study on the prognosis of NPC with PLN metastasis treated with IMRT and found that the hazard ratios (HRs) for distant failure were similar between NPC with initial PLN metastasis and N3 disease. Nevertheless, the small sample size of only 10 NPC patients without PLN‐sparing IMRT was a major limitation of their study, and thus, additional research was needed to confirm their findings. Our previous study revealed that the application of PLN‐sparing IMRT for patients with NPC was not associated independently with an adverse prognosis and those patients typically had PLN with a minimal axial diameter of <10 mm on magnetic resonance imaging (MRI) 17. Consequently, a study following all patients meeting the diagnostic standards for PLN metastasis, based on follow‐up MRI, was needed to further investigate the prognosis of NPC with PLN metastasis.

In this study, a large cohort of NPC patients with PLN metastases treated with IMRT based on follow‐up MRI were retrospectively enrolled. We aimed to assess the effect of PLN metastasis of the prognosis of NPC through a propensity score matching (PSM) study 18, to investigate the appropriate staging category for LN metastasis in the current staging systems for NPC, and finally to provide a reference for treatment.

## Materials and Methods

### Patients and pretreatment evaluation

Records for 1616 consecutive cases of newly diagnosed, pathologically confirmed NPC without distant metastasis that were treated with IMRT in Fujian Provincial Cancer Hospital from June 2005 to December 2011 were retrospectively reviewed. All patients received MRI scans of the nasopharynx and neck. Among the whole study population, 1211 participants were men, and 405 were women, with the male to female ratio being 3:1. Also, 1023 patients were less than 50 years of age, and the other 593 patients were older than 50 years. Historically, 99% of the patients had World Health Organization (WHO) type II or III disease, and only 1% had WHO type I disease. Of the 1616 patients, 45 (2.8%) were found to finally develop PLN metastasis on MRI. The data for other clinical variables including T classification, N classification, and chemotherapy are summarized in Table 1.

**Table 1:** Clinical characteristics of 1616 patients before propensity score matching (PSM) and 90 patients after PSM

Factors	All cases			Matched cases		
	PLN (−) (*n* = 1571)	PLN (+) (*n* = 45)	*P* value	PLN (−) (*n* = 45)	PLN (+) (*n* = 45)	*P* value
Age (years)			0.879			0.827
˃50	576	17		16	17	
≤50	995	28		29	28	
Gender			0.066			0.502
Male	1172	39		41	39	
Female	399	6		4	6	
T classification			0.243			0.965
T1‐2	670	14		14	14	
T3‐4	901	31		31	31	
N classification			0.000			0.964
N0‐1	1118	19		20	19	
N2‐3	453	26		25	26	
Clinical stage			0.003			0.974
I–II	473	4		4	4	
III–IV	1098	41		41	41	
Pathological type			0.594			0.557
WHO I	17	0		0	0	
WHO II or III	1554	45		45	45	
Concurrent chemotherapy			0.742			0.673
Yes	729	22		20	22	
No	842	23		25	23	
Induction chemotherapy			0.067			1.0
Yes	1215	40		40	40	
No	356	5		5	5	
Adjuvant chemotherapy			0.057			1.0
Yes	683	26		26	26	
No	888	19		19	19	
Chemotherapy cycles			0.063			0.598
≥3	1011	35		37	35	
<3	560	10		8	10	

All patients completed the pretreatment evaluation including a medical history, physical examination, nasopharyngoscopy, blood biochemistry, nasopharyngeal and neck MRI examination, chest radiography, abdominal ultrasound, and whole‐body bone scanning, and a few patients also underwent positron emission tomography CT (PET/CT) examination at the treating physician's discretion. All patients were restaged according to the seventh edition of the International Union Against Cancer/American Joint Committee on Cancer (UICC/AJCC) staging system.

### MRI scanning

All patients were examined by a 1.5‐T superconducting MRI system (Signa Excite HD, American GE Company, WI), with scanning performed from the temporal lobe to the middle of the thoracic inlet. Seven scan sequences were performed including axial fat‐suppressed proton density‐weighted imaging (Axial PDWI fs), T2‐weighted coronal short T1 inversion recovery (Cor T2WI‐STIR), diffusion‐weighted imaging (DWI), axial T1‐weighted fat‐suppressed fast spin echo enhanced MRI (Axial T1WI FSEfs + C), and coronal T1‐weighted fast from enhanced scanning spin echo fat suppression (Coronal T1WI FSEfs + C). In the scanning plane, the axial section was vertical to C3, and the coronal section was parallel to C3.

### Imaging assessment and diagnostic criteria

All MRI imaging data were independently reviewed by two head and neck radiology experts. Any disagreements were resolved by consensus. In anatomy, the parotid gland is generally divided into shallow and deep sections through the plane of the facial nerve within the gland. However, it is difficult to display the facial nerve on current MR imaging. As the retromandibular vein is located adjacent to the facial nerve in anatomy, it is clearly shown on MR imaging. Furthermore, Mckean et al. 19 found that the deep PLNs were all closely related to retromandibular vein, most of which were situated lateral to retromandibular vein. Therefore, in this study, the retromandibular vein was used to distinguish the superficial and deep PLN groups on MRI.

At present, no uniform MRI diagnostic criterion for PLN metastasis has been established. The diagnostic criteria for NPC with PLN metastasis in our study were as follows: (1) lymph nodes in level VIII that not only met the diagnostic criteria for cervical lymph node (CLN) metastasis on MRI, but also achieved a partial response (PR) or complete response (CR) according to the Response Evaluation Criteria in Solid Tumors (RECIST) 1.1 criteria 20 by 3 months after definitive radiotherapy, or achieved stable disease (SD) but the node was enlarged on the subsequent follow‐up MRI; (2) preexisting CLNs in level VIII not previously suspected of metastasis but considered recurrent during follow‐up; and (3) PLN metastasis confirmed by fine‐needle aspiration cytology (FNAC) prior to treatment of NPC.

### Treatment

All 1616 patients received definitive IMRT. The details of IMRT in our hospital have been described previously 21. According to the national comprehensive cancer network (NCCN) guidelines, 1404 of 1539 patients with stage II‐IVB disease were given platinum‐based chemotherapy: the sequence given was induction therapy in 275 (19.6%), concurrent therapy in 121 (8.6%), adjuvant therapy in 11 (0.8%), induction‐concurrent therapy in 307 (21.9%), induction‐concurrent‐adjuvant therapy in 282 (20.1%), induction‐adjuvant therapy in 375 (26.7%), and concurrent‐adjuvant therapy in 33 (2.3%).

Salvage treatments such as surgery, chemotherapy, endovascular‐brachytherapy, and external irradiation were provided if a patient had a relapse or persistent disease. After definitive IMRT, 114 patients of the total 1616 patients had residual disease in the local site and 92 patients had regional residuals. For the 114 patients with local residuals, 95 received IMRT as boost treatment (range, 4.5–12.5 Gy), 16 received boost treatment by brachytherapy, 2 received conventional radiotherapy, and 1 patient refused boost treatment. For 92 patients with regional residuals, 80 were treated by IMRT, and the rest were given electronic filed.

### Follow‐up and statistical analysis

Patients were followed up every 3 months for the first 2 years, then every 6 months for the next 3 years, and annually after that. For the entire group, the median follow‐up time was 53 months. The overall survival (OS), local relapse‐free survival (LRFS), regional relapse‐free survival (RRFS), distant metastasis‐free survival (DMFS), and progression‐free survival (PFS) were calculated from the day of first diagnosis to death or last follow‐up, local failure, regional failure, distant failure, and disease failure, respectively. Physical examination and relevant assistant examination (such as nasopharyngoscopy, MRI examination of the nasopharynx and neck, chest radiography, and abdominal ultrasound) were performed during follow‐up.

The SPSS 18.0 software package (SPSS Inc., Chicago, IL) was applied for all statistical analyses. Survival analyses were conducted using the Kaplan–Meier method, and the log‐rank test was used to compare the differences. This study calibrated selection bias and confounding bias using PSM for randomization. The original data were used for logistic regression to calculate propensity scores in the control group and treatment group. In our research, the two groups were divided based on the presence of PLN metastasis by 1:1 matching, according to the following matching factors: gender (male vs. female), age (≤50 years vs. ˃50 years), histological type (I vs. II‐III), T category (T1‐2 vs. T3‐4), N category (N0‐1 vs. N2‐3), concurrent chemotherapy (yes vs. no), induction chemotherapy (yes vs. no), adjuvant chemotherapy (yes vs. no), and chemotherapy cycle (≥3 vs. <3). A *P* value of less than 0.05 was considered statistically significant.

## Results

### Incidence of PLN metastasis and treatment failure patterns

According to our diagnostic criteria, 45 patients were found to have initial PLN metastases, with an incidence of 2.8% (45/1616). Among these cases, the PLNs of 23 patients (51.1%) received a radical prescribed dose of 66–68.2 Gy in 30–31 fractions. Thus, much more than 50% of the ipsilateral parotid volume was irradiated with greater than 26–30 Gy. However, the PLNs of 22 patients (48.9%) were treated by a sparing prescribed dose that was the same as that delivered to the parotid grands. Under such cases, the average mean dose to the ipsilateral parotid gland was 35.4 Gy (range, 33.1–39.1 Gy). Thus, less than 50% of the ipsilateral parotid volume was irradiated with greater than 26–30 Gy. Finally, 19 patients died (13 of distant metastasis, 1 of local recurrence, 2 of treatment complications, and 3 of unknown causes), 9 patients developed disease recurrence (2 with local recurrence and 7 with regional recurrence), and 16 patients developed distant metastasis.

### Association between PLN metastasis and clinical characteristics

For the entire group, PLN metastasis was significantly correlated with N classification (*P *=* *0.001) and clinical stage (*P *=* *0.003). However, the associations between PLN metastasis and other clinical characteristics including T classification, gender, age, pathological type, induction chemotherapy, concurrent chemotherapy, adjuvant chemotherapy, or cycles of chemotherapy were not significant, as shown in Table 1.

### Prognostic value of PLN metastasis

**Figure 1:**
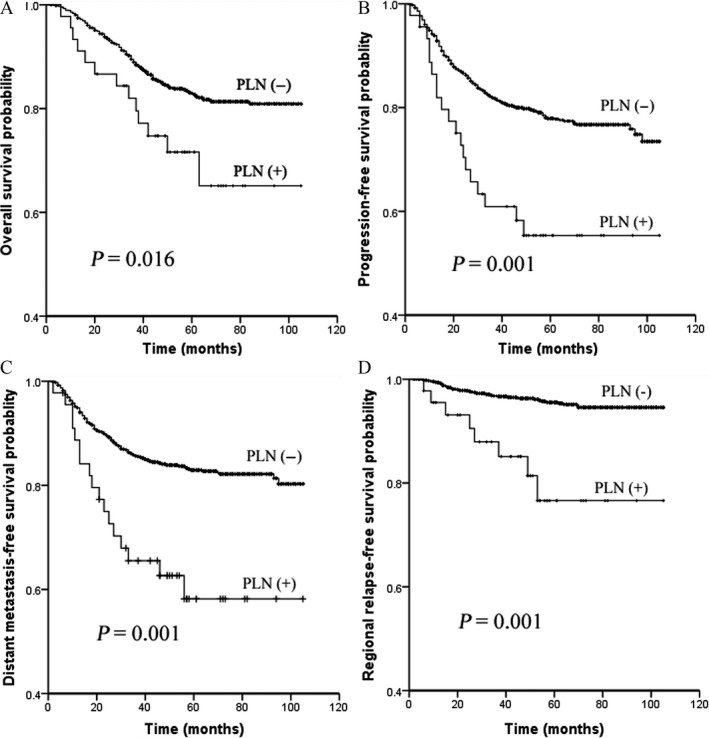
Comparisons of overall survival (A), progression‐free survival (B), distant metastasis‐free survival (C), and regional relapse‐free survival (D) curves between 45 NPC patients with parotid area lymph node (PLN) metastasis and 1571 NPC patients without PLN metastasis.

To investigate whether PLN metastasis was markedly correlated with clinical outcomes, we first compared the survival outcomes between patients with PLN metastasis and those without PLN metastasis by univariate log‐rank survival analysis. Of the 1616 patients, our results showed that patients with PLN metastasis had poorer 5‐year OS, PFS, DMFS, and RRFS compared with those without PLN metastasis (*P *=* *0.016, 0.001, 0.001, and 0.001, respectively), whereas no significant difference in the 5‐year LRFS was found between the two groups (Fig. 1).

**Figure 2:**
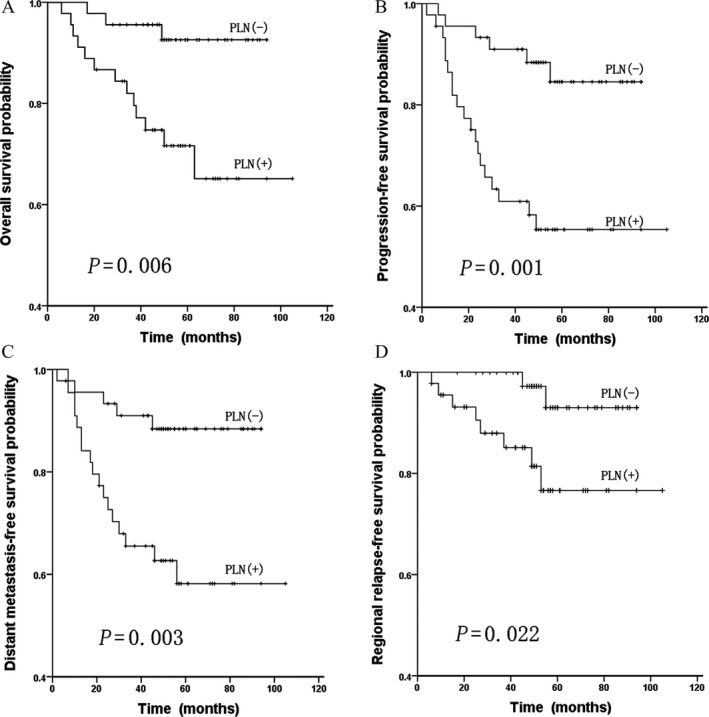
Comparisons of overall survival (A), progression‐free survival (B), distant metastasis‐free survival (C), and regional relapse‐free survival (D) curves between 45 NPC patients with parotid lymph node (PLN) metastasis and 45 patients without PLN metastasis after propensity score matching (PSM).

To further verify the prognostic effect of PLN metastasis, the PSM method was used to calibrate selection bias and confounding bias between the two groups. After matching, no significant differences in various clinical characteristics were observed between 45 patients with PLN metastasis and 45 patients without PLN metastasis, as shown in Table 1. It was found that involvement of PLNs was still a prognostic factor for OS, PFS, DMFS, and RRFS (*P *=* *0.006, 0.001, 0.003, and 0.022, respectively; Fig. 2).

### Comparison of staging categories for PLN metastasis

**Figure 3:**
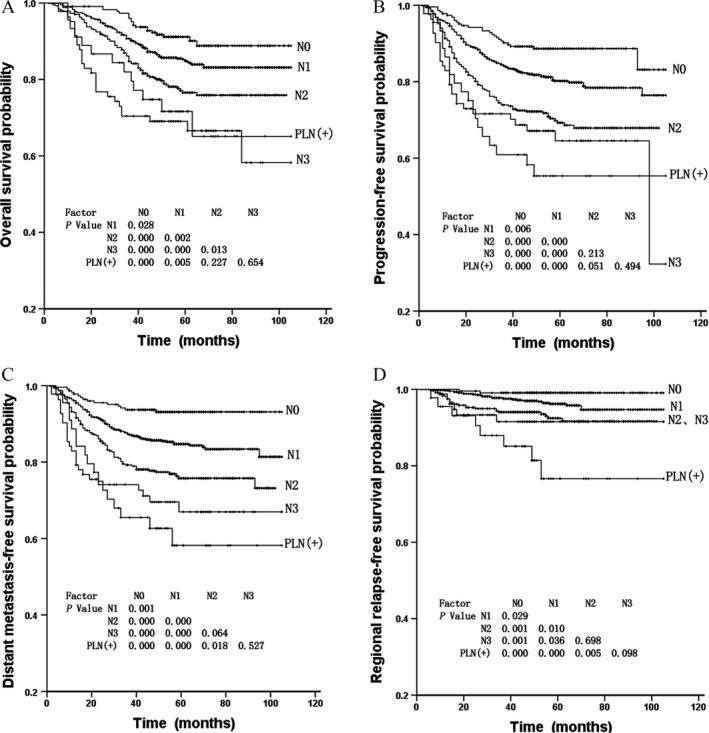
Comparisons of overall survival (A), progression‐free survival (B), distant metastasis‐free survival (C), and regional relapse‐free survival (D) curves between 45 NPC patients with parotid lymph nodes (PLN) metastasis and 1571 patients without PLN metastasis stratified by N classification.

To investigate the prognostic significance of PLN metastasis in the current 7th UICC/AJCC staging system, the 1616 patients with NPC in this study were categorized into five groups: N0 (226 cases), N1 (892 cases), N2 (371 cases), N3 (82 cases), and PLN metastasis (45 cases). As shown in Figure 3, the OS and PFS curves for NPC with PLN metastasis were separated from that for N2 disease, but the differences were not statistically significant (*P *=* *0.227 and 0.051, respectively), and these curves crossed that for N3 disease (all *P *>* *0.05). Nevertheless, the DMFS and RRFS curves for NPC with PLN metastasis were significantly separated from that for N2 disease (*P *=* *0.018 and 0.005, respectively), and also crossed with that for N3 disease (all *P *>* *0.05).

## Discussion

This study represents a novel investigation into the effect of initial PLN metastasis on the prognosis of NPC in a large cohort of patients with NPC in the IMRT era using the PSM method. The incidence of PLN metastasis in NPC was low, but distant metastasis remained the main cause of treatment failure among NPC patients with PLN metastasis. In addition, PLN metastasis of NPC was significantly correlated with N classification and clinical stage, separately. After correcting for selection bias and confounding bias via PSM, our results showed that PLN metastasis was an adverse prognostic factor for OS, PFS, DMFS, and RRFS. Furthermore, the 5‐year DMFS and RRFS rates for NPC with PLN metastasis were inferior to those for N2 disease, whereas they were similar to those for N3 disease. Therefore, the recommended treatment for NPC with PLN metastasis is recommended to be consistent with that for N3 disease to reduce distant metastasis.

Our results showed that the incidence of PLN metastasis was only 2.8%, which was similar to rates in other studies 1, 2, 3, 4, 5, 6.Due to the low frequency of initial PLN metastasis with NPC, little attention has been paid to this condition in clinical practice. To the best of our knowledge, PLN metastasis has not yet been classified in the 7th UICC/AJCC staging system or the Chinese 2008 staging system for NPC. However, once PLN metastasis is diagnosed, it has been generally considered as CLN metastasis by clinical physicians, and then classified according to N classification. In this study, our results indeed revealed that PLN metastasis was significantly related to the N classification and clinical stage, but not to the T classification. This was in line with the finding of Zhang et al. 4. Hence, it is reasonable for PLN metastasis to be included in N classification in clinical practice, and further investigation into the role of PLN metastasis in the current UICC/AJCC staging system is needed.

In the first study of the prognostic significance of PLN metastasis in preliminarily diagnosed NPC patients treated by IMRT, Zhang et al. first found that PLN metastasis was an independent prognostic factor for DMFS in NPC patients and associated with a poor DMFS similar to that for N3 disease 4. Nevertheless, their study included only 10 patients receiving irradiation of the involved PLNs, and this sample size was insufficient for the results to be conclusive. Moreover, the median follow‐up duration in their study was only 38.3 months. In our previous study, we observed that although using PLN‐sparing IMRT could increase the risk of parotid recurrence, it was not an independent prognostic factor for any survival endpoints 16. This may be because patients with NPC who appeared to have recurrence in the periparotid area obtained timely salvage therapy, which contributed to their long‐term survival 12, 13. Therefore, in our current study, 45 patients with PLN involvement, regardless of whether they received radical or sparing irradiation, were enrolled. On univariate analysis, our results showed that NPC patients with PLN involvement had significantly poorer 5‐year OS, PFS, DMFS, and RRFS than those without PLN involvement. To balance the two groups according to different clinical parameters, 45 patients without PLN metastasis were chosen by the PSM method to calibrate confounding bias and selection bias. Then, it was also observed that PLN metastasis was an adverse independent prognostic factor for 5‐year OS, PFS, DMFS, and RRFS in patients with NPC after the PSM method. Therefore, careful attention should be paid to patients with initial PLN involvement.

It is widely accepted that the staging system for cancer is very important as it can be used to accurately guide clinical treatment, assess treatment effect, and predict prognosis 22. To further investigate the prognostic significance of PLN metastasis in the current UICC/AJCC staging system for NPC without distant metastasis, the group of patients with PLN metastasis was divided into subgroups based on N classification for univariate Kaplan–Meier analysis. Our results indicated that the 5‐year OS and PFS rates in cases with PLN metastasis were consistent with those for N2 disease. However, the 5‐year DMFS and RRFS rates for NPC with PLN metastasis were significantly poorer than those for N2 disease but were similar to those for N3 disease. Therefore, PLN metastasis was associated with a similarly poor DMFS as N3 disease, which was in accordance with previous studies 4, 17. Notably, our study demonstrated that distant metastasis was the main cause of treatment failure in NPC patients with PLN metastasis, and a method to effectively reduce distant metastasis in NPC cases with PLN involvement will be pivotal in the advancements of current treatments. Hence, PLN metastasis in patients with NPC should be treated as N3 disease, and more aggressive therapeutic strategies such as more effective systemic chemotherapy should be administered in the same manner as for N3 disease.

Notably, our study had some limitations. First, the 45 patients with NPC and initial PLN metastasis were diagnosed mainly based on the follow‐up MRI, and not on nodal FNAC except in only one case, which may have resulted in the inclusion of false positives. Therefore, in NPC patients, suspected PLN metastases should be confirmed via nodal pathology to accurately guide clinical treatment. However, some parotid lesions are too small to be sampled via FNAC, and other test and studies such as PET/CT are recommended to be performed. Secondly, concurrent chemotherapy has been repeatedly proven to significantly improve the survival prognosis of patients with locally advanced NPC 23, 24, 25, but only 46.5% of 1616 patients in our study received concurrent chemotherapy. This could be due to the fact that some patients were invited to take part in randomized clinical trials to receive certain chemotherapy regimens, or some chemotherapy regimens were given at the discretion of attending physicians according to individual cases. Hence, an increased number of NPC patients with PLN metastasis treated with concurrent chemoradiotherapy is needed to further verify the effect of PLN metastasis on the prognosis of NPC. Finally, this study was a retrospective study, and a relatively small cohort of patients with NPC and PLN metastasis was enrolled due to its low incidence. Thus, a large, prospective, and multicentered case–control study is needed.

## Conclusions

In conclusion, initial PLN metastasis in NPC was significantly associated with N classification and clinical stage. And PLN metastasis was proven to be an adverse prognostic factor for OS, PFS, DMFS, and RRFS in patients preliminarily diagnosed with NPC. In addition, PLN metastasis in NPC patients was correlated with a poor DMFS similar to that for N3 disease. Thus, more aggressive systemic chemotherapy should be used in the same manner as for N3 disease to reduce distant metastasis.

## Conflict of Interest

The authors declare that they have no conflict of interest.
